# A novel plasmonic metal–semiconductor–insulator–metal (MSIM) color sensor compatible with CMOS technology

**DOI:** 10.1038/s41598-023-41346-4

**Published:** 2023-08-28

**Authors:** A. Beheshti Asl, H. Ahmadi, A. Rostami

**Affiliations:** 1 Photonics and Nanocrystals Research Lab (PNRL), Faculty of Electrical and Computer Engineering, University of Tabriz, Tabriz, Iran; 2 SP-EPT Lab., ASEPE Company, Industrial Park of Advanced Technologies, Tabriz, Iran

**Keywords:** Engineering, Optics and photonics

## Abstract

Color detection is one of the top interests in both biological and industrial applications. Specifically, the Determination of the light wave characteristics is vital in photonic technology. One of the features in the color sense that should be found out is its wavelength or color. In this work, we propose a structure that can be used to detect RGB colors separately in an efficient way. The proposed detector consists of the plasmonic filter sensing desired wavelength (red, green, and blue) and the PN diode to convert the received photons to the electrical current. At the input intensity of 1 mW × cm^−2^, the current density for blue, green, and red colors are 27, 35, and 48 µA × cm^−2^, respectively. It is shown that the intensities needed to obtain the current densities of 0.1 µA × cm^−2^ are 3.94, 2.98, and 2.25 µW × cm^−2^ for the blue, green, and red spectra respectively. It should mention that by using high-precision photodetector structures such as PIN diode, the minimum detectable level can be decreased. Simple adjusting for desired wavelength and linear operation for different input intensities are the characteristics of the designed structure. This detector is compatible with CMOS technology and can be easily utilized in numerous applications, such as charge-coupled devices, displays, and cameras.

## Introduction

Since photonic technology’s appearance, designing an efficient detector has been of great interest to researchers. Photodetectors are such devices in which the intensity of incident light is converted to an electrical current. Generally, this conversion is sensitive to the wavelength of incident light. Infrared (IR) and visible light detectors (VLDs) have myriad applications in photonic-based issues such as medical and military imaging, optical communication, and modern cameras^1–8^. The electromagnetic spectrum between 400 and 700 nm is called visible light that should be detected through VLDs. Detection of red, green, and blue (RGB) colors separately and efficiently is the basic duty of VLDs. In other words, color filtering has to be performed in these detectors.

Color detection is the base function of image-sensing devices such as CMOS-based ones^9–11^, and multicolor holograms^12^. Color filters based on pigments and dyes have traditionally been used in organic light-emitting devices (OLEDs) and liquid crystal displays (LCDs)^13,14^. These filters are not sufficiently reliable because organic materials have low chemical stability^11^. Moreover, the organic filtering materials are incompatible with the integration processes^11^. Making use of metamaterials, nanowire waveguides, quantum dots, and plasmonics are the alternatives to designing color filters^15–18^. In the plasmonic phenomenon, the surface resonance at the metal–insulator interface, called surface plasmon resonance (SPR), can be utilized to design a multilayer structure to trap a desired wavelength and act as a filter^19–21^. The simple implementation of plasmonic structures leads researchers to use plasmonics in broad applications such as waveguiding, optical sensing, absorbers, and filters^22–25^. From a filtering point of view, the plasmonic structure can be easily adjusted by the thickness of the insulator layer to change the resonance frequency and, subsequently, the filtered spectrum^26–28^.

Plasmonic filters can be mainly divided into two types: static and dynamic^29^. In contrast to the static case, the dynamic one shows different characteristics depending on the polarization of the incident light^30,31^, heat, or mechanical stress applied to the device^32–34^. Grating, periodic, subwavelength, and hybridized nanoholes^35–37^, and nanodisk arrays^17,37^ are some examples introduced for static filters. The durability and resolution of the plasmonic filters are better than the non-plasmonic ones. For this reason, we have used the plasmonic-based structure to design the color filter^29,38–40^.

In this paper, we propose a multilayer plasmonic photodetector that includes three isolated parts related to RGB colors. Each part generates a current, connected with the light intensity at the corresponding wavelength. Furthermore, because of the filtered wavelength's dependency on the semiconductor layer’s thickness that can be realized by lithography, any requested wavelength at the visible spectrum can be achieved. The finite difference time domain (FDTD) simulation of the designed structure is carried out to demonstrate the transmission spectrum. The current densities generated in the PN diode associated with each color are obtained afterward. It is expected that utilizing the more efficient photodiode instead of simple PN results in better functions. This capability makes the proposed device compatible with the existing planar technology. Moreover, the current dependency on the incident light intensity, the type of oxide layer (SiO_2_ and Si_3_N_4_), and the detector dimensions are investigated.

## The proposed structure

**Figure 1:**
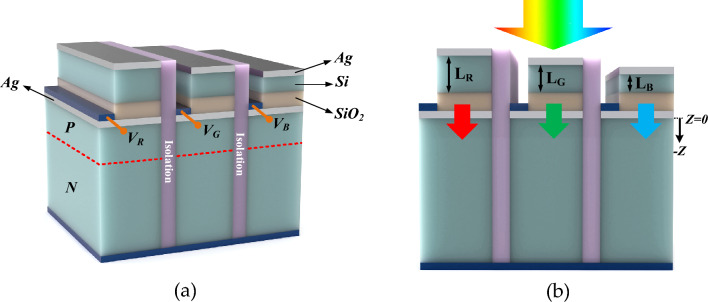
(**a**) 3D view of the device. V_R_, V_G_, and V_B_ are the bias voltages applied to the contact in the red, green, and blue regions. (**b**) 2D view of the device. The silver and silicon dioxide layers’ thickness is 10 and 50 nm for all regions. The thickness of the silicon layers for the red, green, and blue regions are L_R_ = 100 nm, L_G_ = 70 nm, and L_B_ = 50 nm, respectively. The thickness of the diode is 10 µm.

The device is designed to detect some of the predefined wavelengths in the visible spectrum. The schematic of the structure (color filter) is depicted in Fig. 1. It consists of three isolated parts for red, blue, and green colors. Each part is mainly divided into the filtering section and the photodiode. The filter results in the maximum transmission at the desired wavelength, and then the photodiode generates the current at the relevant wavelength. The filter is a metal–semiconductor–insulator–metal (MSIM) structure. Silver and silicon are the metal and semiconductor parts, respectively. The insulator layer can be silicon oxide or silicon nitride. The thickness of the sliver and insulator layer are 10 and 50 nm. However, the thickness of the silicon layer depends on the wavelength that should be detected. Herein, the silicon is determined at 50, 70, and 100 nm for blue, green, and red, respectively. The photodiode includes simple silicon PN diode with a thickness of 10 µm.

## Simulation method

At first, the finite difference time domain (FDTD) method is used to evaluate the transmission spectrum of the proposed device by solving Maxwell’s equations. This spectrum is utilized to calculate the absorption via integrating absorption spectra using A(r) = ∫A(r, ω)dω. Position-dependent absorption, A(r,ω), is given by Eq. 1 where E(r, ω) is the total electric field, including the incident and scattered fields, Im ε(r, ω) is the imaginary part of permittivity and Pin is the power of input intensity^41^.

1 $$\mathrm{A}(\mathrm{r},\upomega ) =\frac{\upomega {\upvarepsilon }_{0}}{2} \frac{\mathrm{Im}\left(\upvarepsilon \left(\mathrm{r},\upomega \right)\right){\left|\mathrm{E}(\mathrm{r},\upomega )\right|}^{2}}{{\mathrm{P}}_{\mathrm{in}}}$$

The generation rate is necessary to obtain the current density. The total generation rate is calculated by G(r) = ∫g(r, ω)dω in which G and g are the total and frequency-dependent generation rates, respectively. The generation rate at the specific frequency is obtained by Eq. (2) where Pabs is the absorbed power^41,42^.

2 $$\mathrm{g}\left(\mathrm{r},\upomega \right)=\frac{{\mathrm{P}}_{\mathrm{abs}}}{\mathrm{\hslash \omega }}=\frac{{\upvarepsilon }_{0}}{2\mathrm{\hslash }} \frac{\mathrm{Im}(\upvarepsilon (\mathrm{r},\upomega )){\left|\mathrm{E}(\mathrm{r},\upomega )\right|}^{2}}{{\mathrm{P}}_{\mathrm{in}}}$$

Finally, The Poisson, continuity, and drift–diffusion equations represented in Eqs. (3), (4), and (5), respectively, are solved in the diode self-consistently by applying the obtained generation rate and using the finite element method.

3 $$\frac{{\partial }^{2}V}{ {\partial }^{2}z}=-\frac{\partial E}{\partial z}=-\frac{\rho }{\varepsilon }=\frac{q}{\varepsilon }[p-n+{N}_{D}\left(x\right)-{N}_{A}(x)]$$

4-1 $$\frac{{\partial }^{2}{J}_{n}}{{\partial z}^{2}}+G-R=0$$

4-2 $$-\frac{{\partial }^{2}{J}_{p}}{{\partial z}^{2}}+G-R=0$$

5-1 $${J}_{n}=ep{\mu }_{n}E+e{D}_{n}\frac{{\partial }_{n}}{{\partial }_{z}}$$

5-2 $${J}_{p}=ep{\mu }_{p}E-e{D}_{p}\frac{{\partial }_{P}}{{\partial }_{z}}$$

In Eq. (3), N_D_ and N_A_ are the density of ionized donor and acceptor atoms, respectively. The generation rate mentioned above is used in the continuity equations. In this equation, R represents the recombination rate. In the drift–diffusion equations that result in current densities, μ_n_, μ_p_, D_n_, and D_p_ are electron and hole mobilities and their diffusion coefficients, respectively^43^.

The silicon layer of the filter is considered intrinsic; however, in the diode, the doping of the P and N are assumed 2 × 10^16^ cm^−3^ and 2 × 10^17^ cm^−3^, respectively. Moreover, the mobilities are considered 1471 and 470.5 cm^2^/V s for electron and hole, respectively. Furthermore, the incident visible light is supposed to be the plane wave.

## Results and discussion

**Figure 2:**
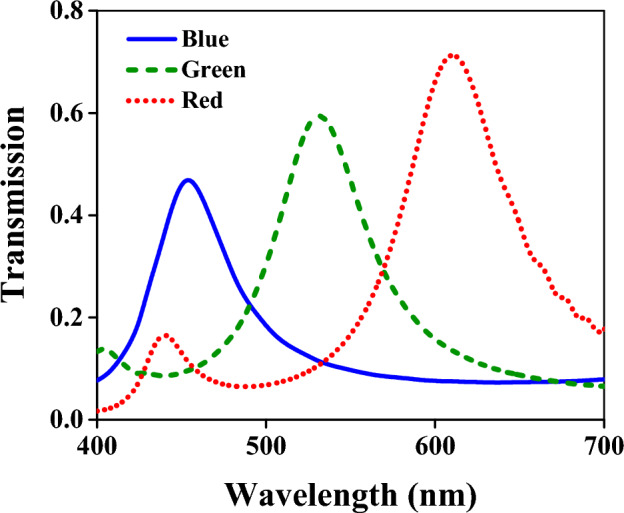
The transmission spectra for a device with the SiO_2_ layer. The solid blue line corresponds to the transmission just below the part of the structure related to the blue color. Similarly, the dashed green and dotted red lines represent the transmissions in which the output waves are monitored above the PN diode exactly below the respective region.

The transmitted light has been evaluated below every part of the structure corresponding to RGB colors to investigate the operation of the device from a transmission point of view. Figure 2 demonstrates the transmission spectra of the device in the visible band. The solid blue, the dashed green, and the dotted red line correspond to the transmission in which the output wave has been monitored separately below the part of the structure designed for blue, green, and red, respectively. The peak frequencies and related quality factors are given in Table 1. In the blue region, the maximum value of the transmission is 0.47 at the wavelength of 450 nm. This maximum emerges at 530 and 610 nm wavelengths for the green and red parts of the spectrum with values of 0.6 and 0.71, respectively. The quality factors for blue, green, and red spectra are 7.5, 7.91, and 7.92, respectively. The difference in amplitude of the output light at RGB colors occurs due to the refractive index's dependency on the wavelength. The obtained spectra are analogous to CIE 1931; hence the device is substantially suitable for cameras and artificial eyes^44^. Moreover, in every RGB region, the peak wavelength can be easily changed by the adjustment of the dimension of the silicon layer.

**Table 1:** Peak wavelengths and quality factors for RGB spectra.

Spectrum	Central wavelength (nm)	Quality factor
Blue	450	7.5
Green	530	7.91
Red	610	7.92

The color filter's crosstalk, an important parameter for using the filter as an image sensor, has been calculated by Eqs. 6, 7, and 8 for the blue, green, and red spectra, respectively^45^. T_B_, T_G_, and T_R_ represent the transmissions for the blue, green, and red regions. The calculated crosstalks are given in Table 2. The colors mentioned in the rows and columns refer to the reference and overlapping spectra inserted in the denominators and numerators of Eqs. (6), (7), and (8), respectively.

**Table 2:** Crosstalks for the color filter.

Reference spectrum	Blue	Green	Red
Blue	–	0.306	0.298
Green	0.249	–	0.261
Red	0.127	0.244	–

6 $${C}_{B,i}=\frac{{\int }_{420nm}^{480nm}d\lambda {T}_{i}}{{\int }_{420nm}^{480nm}d\lambda {T}_{B}},i=G,R$$

7 $${C}_{G,i}=\frac{{\int }_{500nm}^{560nm}d\lambda {T}_{i}}{{\int }_{500nm}^{560nm}d\lambda {T}_{G}},i=B,R$$

8 $${C}_{R,i}=\frac{{\int }_{580nm}^{640nm}d\lambda {T}_{i}}{{\int }_{580nm}^{640nm}d\lambda {T}_{R}},i=B,G$$

**Figure 3:**
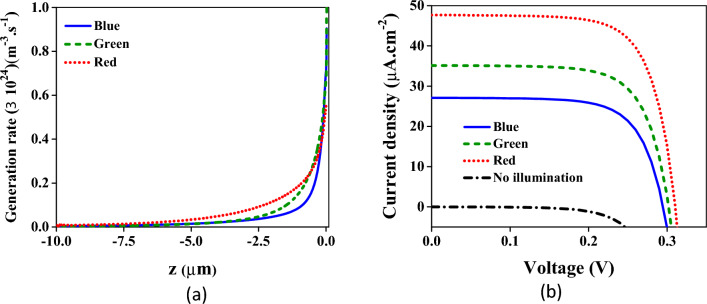
(**a**) Position-dependent generation across the Diode. Solid blue, dashed green, and dotted red lines are the generation obtained at the end of the three regions of the filtering part corresponding to RGB colors. (**b**) Current densities for RGB colors versus the bias voltage. The blue solid line is the current density given in the contact of V_B_. The densities in the contacts of V_G_ and V_R_ have been demonstrated by green dashed and dotted red lines, respectively. Without illumination, the black dash-dotted line is obtained in which the current density is roughly zero in all contacts.

Absorption has been obtained using the equations mentioned in the previous section. Consequently, the position-dependent generation has been acquired for every color, depicted in Fig. 3a. Herein, z represents the position of the diode from z = − 10 um to z = 0. As expected, the generation is maximum at z = 0 and exponentially decreased due to the absorption reduction in the diode down to the common contact. Applying this generation to the continuity equation and utilizing the drift–diffusion equations result in current densities demonstrated in Fig. 3b. This figure shows the current densities given by three contacts of the structure. The solid blue line is the current density in the contact V_B_ (Fig. 1) related to the region in which the silicon layer is 50 nm. Similarly, the dashed green and dotted red lines are current densities in the contacts V_G_ and V_R_, respectively. Furthermore, the black dash-dotted line shows the current density where the device has not been illuminated, and for this reason, the density is approximately zero in all contacts. As is clear, the higher transmission for the red color leads to higher total generation, resulting in further current density compared to the blue and green colors.

**Figure 4:**
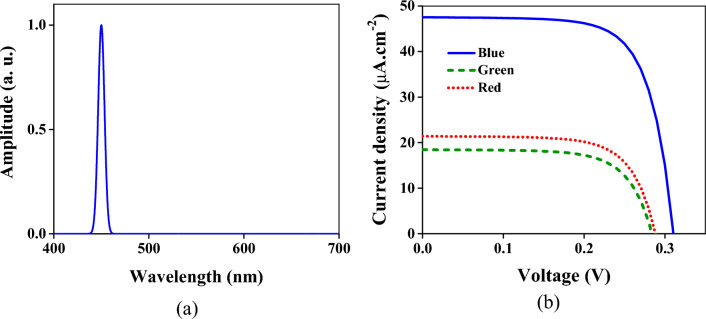
(**a**) The incident wave spectrum is a Gaussian beam with a center frequency of 450 nm and bandwidth of 20 nm. (**b**) The obtained current densities from three regions of the structure. The blue solid, green dashed, and red dotted lines are the current densities of the respective regions.

The structure has been illuminated by the wave containing the part of the visible spectrum for a further qualitative evaluation of the crosstalk. At first, the spectrum between 440 and 460 nm (within the blue spectrum) depicted in Fig. 4a has been applied to the device. In this situation, the obtained current densities are shown in Fig. 4b. As is seen, the current given by the V_B_ contact is more than others. The current density in the contact related to the blue color is 27.1 µA × cm^−2^; however, it is 35.1 and 47.7 µA × cm^−2^ for the green and red colors. The intensity required to obtain the current density of 0.1 µA × cm^−2^ has been acquired as a criterion. This intensity for the blue, green, and red regions is 3.94, 2.98, and 2.25 µW × cm^−2^, respectively.

**Figure 5:**
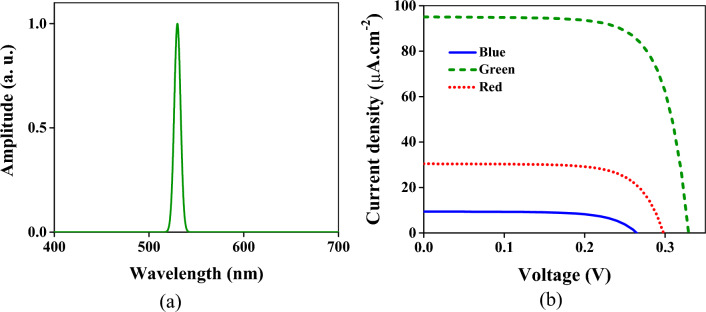
(**a**) The incident wave spectrum is a Gaussian beam with a center frequency of 530 nm and bandwidth of 20 nm. (**b**) The obtained current densities of three regions of the structure. The blue solid, green dashed, and red dotted lines are the current densities of the respective regions.

Applying the spectrum between 520 and 540 nm (Fig. 5a) to the color filter results in the current densities depicted in Fig. 5b, in which the green region represents the current density of 97 µA × cm^−2^ that is at least three orders more than other regions. These results indicate that the absorption share of the blue and red regions is significantly less than the green part.

**Figure 6:**
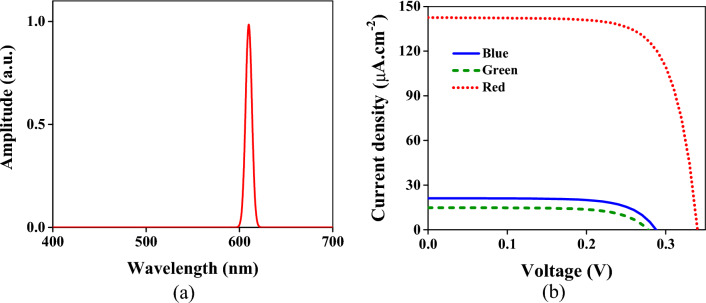
(**a**) The incident wave spectrum is a Gaussian beam with a center frequency of 610 nm and bandwidth of 20 nm. (**b**) The obtained current densities of three regions of the structure. The blue solid, green dashed, and red dotted lines are the current densities of the respective regions.

For evaluation of the red region of the color filter, the input spectrum of light is considered between 600 and 620 nm shown in Fig. 6a. The obtained current densities demonstrated in Fig. 6b show that the current in the red region is 147 µA × cm^−2^. This value is approximately six orders more than the current density of the blue region and seven orders more than the red one.

**Figure 7:**
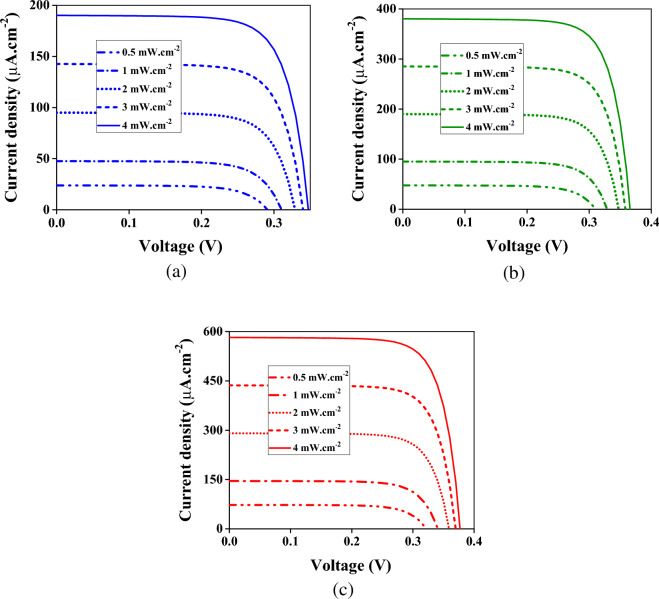
The current densities at different input intensities in the blue (**a**), green (**b**), and red (**c**) contacts. (**a**) The incident wave spectrum is a Gaussian beam with a center frequency of 450 nm and bandwidth of 20 nm (Fig. 4a). (**b**) The incident wave spectrum is a Gaussian beam with a center frequency of 530 nm and bandwidth of 20 nm (Fig. 5a). (**c**) The incident wave spectrum is a Gaussian beam with a center frequency of 610 nm and bandwidth of 20 nm (Fig. 6a).

**Figure 8:**
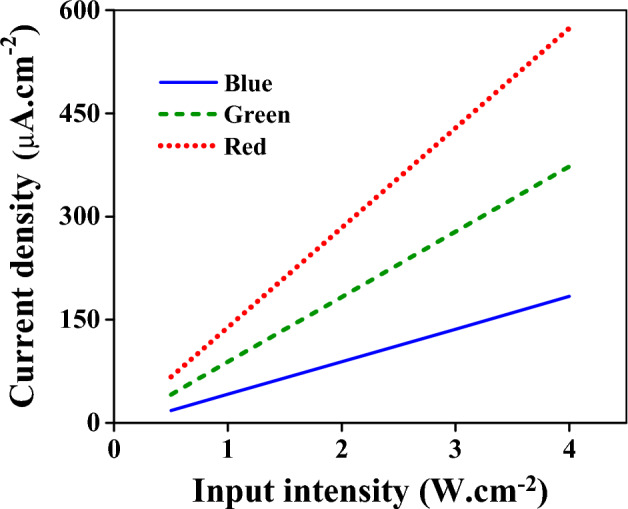
The current density versus input intensity at 0.25 [V]. The solid blue line is for the input spectrum of 430 to 460 nm. The green dashed line and red dotted line are obtained when the input spectrum is 520 to 540 nm and 600 to 620 nm, respectively.

The Gaussian beams shown in Figs. 4 and 5 with different intensities are applied to the device to investigate the effect of input intensity on the current density. The results are illustrated in Fig. 7. The higher the input intensity, the more the current density. In Fig. 7a, the blue spectrum between 430 and 460 nm with intensities of 0.5, 1, 2, 3, and 4 mW × cm^−2^ are applied to the color filter. It leads to the maximum current densities of 18.1, 41.7, 88.9, 136, and 184 µA × cm^−2^, respectively. When the input intensity is within the green spectrum (520 to 540 nm), the maximum current densities are 41.17, 89, 183, 278, and 373 µA × cm^−2^ for the so-called respective input intensities. The higher transmitted intensity in the red spectrum results in the current intensities higher than the green and blue spectra. The maximum current densities of 66.9, 139, 284, 429, and 574 µA × cm^−2^ are obtained for input intensities of 0.5, 1, 2, 3, and 4 mW × cm^−2^, respectively, when the input spectrum is between 615 and 635 nm. As is seen from Fig. 7, the current density is linear concerning the input intensity. Figure 8 demonstrates this linear operation better. In this figure, the current densities are depicted at the 0.25 [V] versus input intensities when the electric field is considered Gaussian within the mentioned blue, green, and red spectrums.

**Figure 9:**
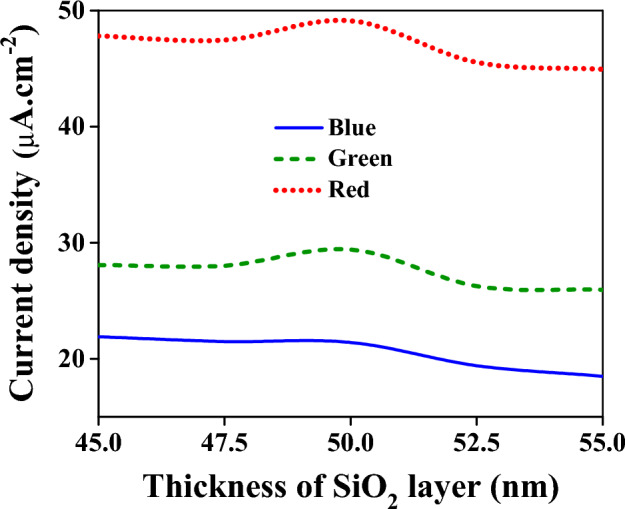
Current density for RGB colors in terms of the SiO_2_ layer’s thickness.

As mentioned previously, the thickness of the SiO_2_ layer is 50 nm. The change in this dimension may result in an alteration of the current density; hence, an investigation of the influence of the SiO_2_ layer’s thickness has been done, and the results are depicted in Fig. 9. As is seen, the best dimension of the SiO_2_ layer is 50 nm leading to the maximum current densities for all three colors even though the changing of current densities is negligible for thicknesses between 45 and 55 nm. Therefore, in device fabrication, a few nanometer variances in SiO_2_ thickness are insignificant.

**Figure 10:**
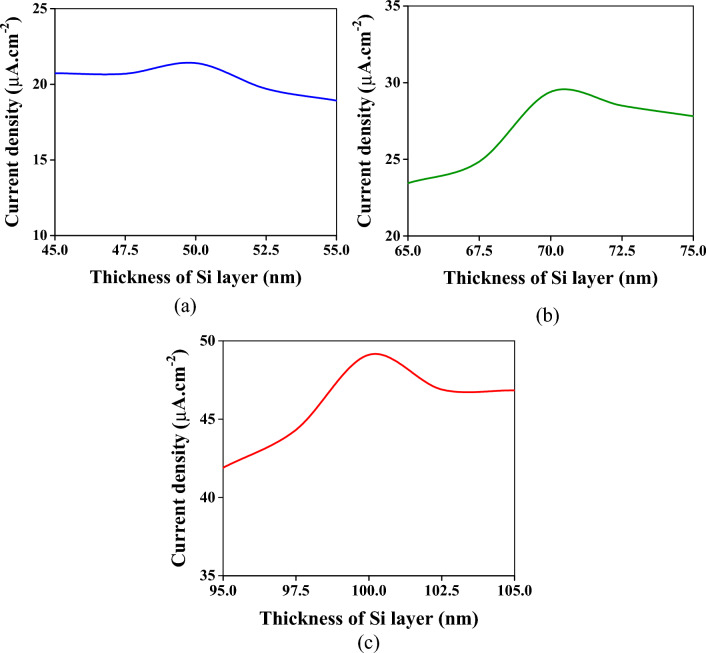
The effect of change in the thickness of the Si layer on the current densities. The alteration is considered to be done around the selected dimensions which are 50 nm, 70 nm, and 100 nm for the blue, green, and red spectrums, respectively. (**a**) For the blue spectrum. (**b**) For the green spectrum. (**c**) For the red spectrum.

Another crucial dimension in the filter operation is the dimension of the Si layer. This layer determines which wavelength can be transmitted with a minimum reflection. Similar to the SiO_2_ layer, the thickness of the Si layer has been altered to evaluate its effect on the current densities. In the blue region, as is clear from Fig. 10a, the influence of this alteration around the selected value (50 nm) is negligible. For the green and red colors, the change in the Si layer results in small changes in the current densities, as demonstrated in Fig. 10b,c. For these reasons, the proposed device is reliable from the fabrication point of view.

**Figure 11:**
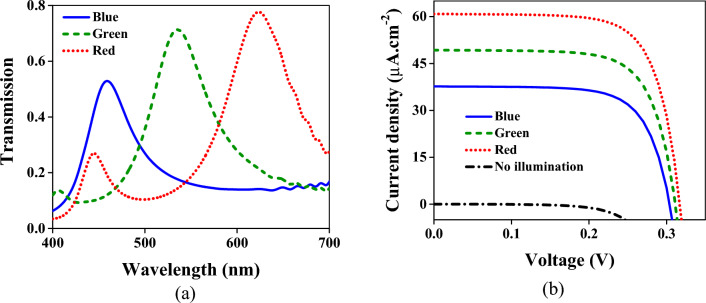
(**a**) The transmission spectra when the SiO_2_ layer is replaced with the Si_3_N_4_. The solid blue line corresponds to the transmission just below the part of the structure related to the blue color. Similarly, the dashed green and dotted red lines represent the transmissions in which the output waves are monitored above the PN diode exactly below the respective region. (**b**) Current densities for RGB colors versus the bias voltage. The blue solid line is the current density given in the contact of V_B_. The densities in the contacts of V_G_ and V_R_ have been demonstrated by green dashed and dotted red lines, respectively. Without illumination, the black dash-dotted line is obtained in which the current density is roughly zero in all contacts.

For better operation, the SiO_2_ layer can be replaced with another insulator, such as the Si_3_N_4_. The thickness of the Si_3_N_4_ layer is considered 50, 70, and 100 nm for the blue, green, and red spectra, respectively, which are precisely the thicknesses of the SiO_2_ layers. This replacement led to the transmission spectrums in Fig. 11a. Comparing this figure with Fig. 2 indicates that the operation of the device is promoted. The transmission value for all colors is better than before. Higher transmission value causes higher current densities demonstrated in Fig. 11b. The maximum current densities for the blue, green, and red regions are 37.7, 49.3, and 60.9 µA × cm^−2^, respectively.

## Conclusions

The color filter based on the plasmonic multilayer structure was proposed in which three isolated parts were designed to detect three RGB colors separately. Every part consists of two main sections: filtering and diode. The filtering includes The SiO_2_ or insulator, The Si, and two silver layers. The thickness of silver and insulator are 10 and 50 nm, respectively. However, the Si layer’s thickness is considered 50, 70, and 100 nm for the respective blue, green, and red colors. The diode was considered a simple PN. For better operation, more sophisticated structures can be used for the diode. The device was simulated to obtain the transmission spectra for three RGB regions. Continually, the generation and current densities related to every color were obtained using the transmission. Furthermore, the effect of the insulator and Si layer’s thickness on current densities was investigated. Finally, the SiO_2_ layer was replaced with the Si_3_N_4_ to show that the operation of the device can even be promoted. The current densities increase approximately 40 percent for blue and green and 10% for red.

## Data Availability

All data generated and analysed during the current study are available from the corresponding author on reasonable request.
